# Health Policy, Planning, and Management: reflections based on CSP’s experience

**DOI:** 10.1590/0102-311XEN164524

**Published:** 2024-10-18

**Authors:** Luciana Dias de Lima, Mônica Martins

**Affiliations:** 1 Escola Nacional de Saúde Pública Sergio Arouca, Fundação Oswaldo Cruz, Rio de Janeiro, Brasil.

Studies aiming at understanding and contributing to the policies, organization, and management of health systems and services form one of the structuring axes of Public Health, both in the international and national scenarios. Such studies integrate an area with a wide variety of themes and applications, at multiple levels and dimensions ^1^, encompassing the broader level of public policy formulation and implementation, including health systems configuration, to the level of interpersonal relationships established in work routine and healthcare. Moreover, it involves the development of strategies to improve equity in access, use, and effectiveness of health services, as well as the production and evaluation of technologies and the allocation of financial resources.

In Brazil, this area gained momentum by the late 1970s, associated with the movement for redemocratization and establishment of the Brazilian Unified National Health System (SUS, acronym in Portuguese). This involved collaboration between academics from different disciplinary fields, healthcare providers, and social movements ^2^. Gradually, the area has consolidated itself as one of the three subfields that make up Public Health, called Health Policy, Planning, and Management (PPGS, acronym in Portuguese), together with Epidemiology and the Social Sciences and Humanities in Health ^3^.

*Cadernos de Saúde Pública* (CSP) expresses the dynamics and evolution of this subfield, which has been present in all the moments that mark CSP’s 40 years of scientific publications ^4^. An analysis of the titles from 834 articles published from 2001 to June 2024, evaluated by Associate Editors linked to PPGS, reveal a diversity of themes, approaches, and methods. While acknowledging that overlaps with other subfields of Public Health are both possible and desirable ^5^ ^,^ ^6^, some thematic focuses predominated in this period, such as evaluation, primary care, medications, health policies, access to healthcare, specialized care, and health information. Moreover, the implementation of policies at the national, regional, and local levels, or aimed at the health needs, known or emerging, are mirrored in the scientific production in CSP.

However, variations regarding themes and approaches have occurred over time. These changes are not only due to the diversification of the representativeness of the PPGS in the journal’s Editorial Board, but also to its distinctive characteristics, present in the choice of objects, theoretical references, and study design. Among the peculiarities of this subfield ^7^, the association between academia, health policies, and interventions stands out, as well as the influence of the government agenda and the issues affecting health systems and services in shaping research topics and questions. Research on PPGS addresses both the causes of the investigated phenomena and the repercussions of policies and the health care model on them. There is a certain eclecticism in the choice of theoretical and methodological references and a preference for recent time frames in the investigations.

Maintaining the social and political relevance of the studies developed, ensuring the quality and methodological rigor of the research, and promoting the dissemination and applications of the knowledge produced are permanent challenges in this subfield, which go beyond the public health agenda of many countries. In the Brazilian context, it must be emphasized the importance of internationalizing scientific production, expanding the dialogue between different epistemological perspectives, especially between countries in the Global South.

Another challenge refers to the need to further strengthen the integration between academic research and the professional practice of health management, maintaining the critical perspective and the quality of scientific publications. In Brazil, the expansion of master’s and doctoral programs highlights the importance of PPGS in academic graduate education in Public Health due to the connection between these programs and the sphere of health policies and services. Therefore, researchers and managers must be trained to move between these two worlds, contributing to the construction of health policies and management practices that value science for its capacity to produce knowledge, while also being sensitive to local realities and social demands.

The scientific production of Public Health as a whole has been challenged by the health needs arising from the transformations of the contemporary world ^8^. In this context, some emerging themes for PPGS authors are still poorly represented in CSP publications. The associations established between the State, market, and society in the creation of public policies to address these changes - diverse in nature and impact countries and social groups unevenly - exemplify an approach that can be explored by the subfield. How various markers of social inequalities (such as class, gender, sexuality, skin color/race, ethnicity, accessibility, and housing) interact with each other, conditioning health needs, access, use, and outcomes of health policies and services, is another theme that must be thoroughly investigated. Not to mention issues related to climate change and health emergencies, which require resilience from healthcare systems and new approaches for anticipating and preparedness. These approaches should always be aligned with sustainability and equity, based on comprehensive, fast, and reliable data to generate evidence to support decision-making, especially in times of rapid technological and informational shifts ^9^.

Research efforts directed to these emerging themes will require greater collaboration between different fields of knowledge. Valuing a systemic and interdisciplinary approach ^10^, so alluded to in current times, requires collaboration between researchers with different traditions and specializations within the subfield, as well as with those from Epidemiology, Social Sciences and Humanities in Health backgrounds, and other related areas and disciplines.

During the writing of this editorial, we were surprised by the allegations of harassment and the dismissal of Minister Silvio Almeida, who had been at the head of the Brazilian Ministry of Human Rights and Citizenship since January 2023 ^11^. The case refers both to human rights and public health issues, as well as to feminist and anti-racist struggles, and can be used to exemplify the potential of studies in the PPGS subfield, in its interface with other spheres of science, to deal with complex issues.

In this scenario, with a focus on politics and institutional management, research can contribute to in-depth analyses of the contexts related to situations of moral or sexual harassment experienced by women in organizations, identifying their repercussions and interactions with society. Such an understanding allows us to unveil the power dynamics, the interests involved, and the factors that facilitate or hinder the implementation of effective policies. Scientific studies can suggest recommendations for the improvement of internal practices of prevention, detection, and response to these cases, which promote healthy and safe work environments. Furthermore, it highlights the need for transparency and accountability in investigation processes and institutional decisions, enabling them to be conducted ethically and fairly. The dissemination of scientific information that supports victims’ rights and reinforces the responsibilities of public managers is also an essential aspect. Studies can also indicate intersectoral response and prevention strategies, such as educational programs and awareness campaigns, which not only address harassments, but contribute to transforming the institutional culture, reducing the occurrence of violence experienced by women.

In summary, the PPGS subfield plays a fundamental role in shaping Public Health. Its challenges and contributions are central to the construction of policies and fairer, more effective, and democratic health systems. Greater advances in the scientific production of this subfield will depend on the ability of its researchers and professionals to face the contemporary challenges and promote a continuous dialogue between research and management practice, aiming at improving the living conditions and health of populations.

CSP will always be open to receiving your contributions!
