# Supplementary material for: Health Policy, Planning, and Management: reflections based on CSP’s experience
Source: Cad Saude Publica. 2024 Oct 18;40(10):e00164524. doi: 10.1590/0102-311XEN164524 (PMC11488820; doi:10.1590/0102-311XEN164524)
Supplement: Translation

# Translation: Política, Planejamento e Gestão em Saúde: reflexões a partir da experiência de CSP

**DOI:** 10.1590/0102-311XPT164524

**Authors:** Luciana Dias de Lima, Mônica Martins

**Affiliations:** 1 Escola Nacional de Saúde Pública Sergio Arouca, Fundação Oswaldo Cruz, Rio de Janeiro, Brasil.

Estudos com o objetivo de compreender e contribuir com as políticas, a organização e a gestão dos sistemas e serviços de saúde conformam um dos eixos estruturantes da Saúde Coletiva, no cenário internacional e nacional. Tais pesquisas integram uma área com grande variedade de temas e aplicações, em múltiplos níveis e dimensões ^1^. Desde o plano mais geral da formulação e implementação de políticas públicas e configuração de sistemas de saúde até o plano das relações interpessoais estabelecidas nos processos de trabalho e cuidado em saúde. Envolvendo o desenvolvimento de estratégias para melhorar a equidade no acesso, no uso e na efetividade dos serviços de saúde, até a produção e a avaliação da incorporação de tecnologias e alocação de recursos financeiros.

No Brasil, essa área teve impulso a partir do final dos anos 1970, associada ao movimento de redemocratização e constituição do Sistema Único de Saúde (SUS), que envolveu a articulação entre acadêmicos de diferentes campos disciplinares, profissionais de saúde e movimentos sociais ^2^. Gradativamente, a área se consolidou como um dos três subcampos que conformam a Saúde Coletiva, denominado como Política, Planejamento e Gestão em Saúde (PPGS), juntamente com a Epidemiologia e as Ciências Sociais e Humanas em Saúde ^3^.

*Cadernos de Saúde Pública* (CSP) expressa a dinâmica e a evolução desse subcampo, que esteve presente em todos os momentos que marcam seus quarenta anos de publicação científica ^4^. O exame dos títulos de 834 artigos publicados entre 2001 e junho de 2024, avaliados por Editores Associados vinculados à PPGS, revelam diversidade de temas, abordagens e métodos. Embora reconheça-se que sejam possíveis (e desejáveis) sobreposições com os demais subcampos da Saúde Coletiva ^5^ ^,^ ^6^, alguns núcleos temáticos predominaram nesse período, tais como: avaliação, atenção primária, medicamentos, políticas de saúde, acesso ao cuidado em saúde, atenção especializada e informações em saúde. Ademais, a implementação de políticas, nos âmbitos nacional, regional e local, ou voltadas para as necessidades de saúde, conhecidas ou emergentes, espelham-se na produção científica em CSP.

Entretanto, variações e enfoques em relação às temáticas ocorreram ao longo do tempo. Tais mudanças se devem à diversificação da representatividade do subcampo no Conselho Editorial da revista, mas também às suas características distintivas, presentes na escolha de objetos, referenciais teóricos e delineamento dos estudos. Entre as peculiaridades desse subcampo ^7^, destacam-se a proximidade entre o meio acadêmico, as políticas e intervenções em saúde, bem como a influência da agenda governamental e dos problemas que afetam os sistemas e serviços de saúde, na definição de temas e questões de pesquisa. Há preocupações tanto com as causas dos fenômenos investigados quanto com as repercussões das políticas e do modelo de atenção à saúde sobre eles. Verifica-se certo ecletismo na escolha de referenciais teóricos e metodológicos, assim como a preferência de recortes temporais recentes nas investigações.

Manter a relevância social e política dos estudos desenvolvidos, garantir a qualidade e o rigor metodológico das pesquisas e promover a disseminação e as aplicações dos conhecimentos produzidos são desafios permanentes desse subcampo, que transpassam a agenda da Saúde Coletiva de muitos países. No caso brasileiro, ressalta-se a importância de internacionalizar a produção científica, ampliando o diálogo entre diferentes perspectivas epistemológicas, principalmente entre países do Sul Global.

Outro desafio se refere à necessidade de fortalecer ainda mais a integração entre a pesquisa acadêmica e a prática profissional da gestão em saúde, mantendo a perspectiva crítica e a qualidade das publicações científicas. No Brasil, a expansão dos cursos de mestrado e doutorado, reforça a relevância da PPGS na formação de pós-graduação *stricto sensu* da Saúde Coletiva, pela relação que esses programas estabelecem com o ambiente das políticas e dos serviços de saúde. Isso implica formar pesquisadores e gestores, capazes de transitar entre esses dois mundos, contribuindo para a construção de políticas de saúde e práticas de gestão que valorizem a ciência por sua capacidade de produzir conhecimento, mas também sensíveis às realidades locais e às demandas sociais.

As necessidades de saúde decorrentes das transformações do mundo contemporâneo desafiam a produção científica da Saúde Coletiva como um todo ^8^. Nesse contexto, verificam-se temas emergentes para autores da PPGS, ainda não suficientemente representados em CSP. Relações estabelecidas entre Estado, mercado e sociedade na produção de políticas públicas para responder a essas mudanças, que são de ordem diversa e afetam de modo desigual países e grupos sociais, é um exemplo de enfoque que pode ser explorado pelo subcampo. O modo como diferentes marcadores das desigualdades sociais (tais como classe, gênero, sexualidade, raça, etnia, acessibilidade e moradia) interagem entre si, condicionando as necessidades de saúde, acesso, uso e resultados das políticas e serviços de saúde, é outra temática que merece ser aprofundada. Além de questões relacionadas às mudanças climáticas e às emergências sanitárias, que exigem resiliência dos sistemas e serviços de saúde e novas formas de antecipação e prontidão, em prol da sustentabilidade e equidade. Para essas questões, são imprescindíveis informações abrangentes, ágeis e confiáveis que permitam a produção de evidências para auxiliar a tomada de decisão, sobretudo em tempo de enormes avanços tecnológicos e informacionais ^9^.

Os esforços de pesquisa direcionados para esses temas emergentes exigirão maior articulação entre saberes. A valorização de uma abordagem sistêmica e interdisciplinar ^10^, tão aludida nos tempos atuais, requer colaboração entre pesquisadores com diferentes tradições e especializações no interior do subcampo, bem como com aqueles vinculados à Epidemiologia, às Ciências Sociais e Humanas em Saúde, e às demais áreas e disciplinas afins.

Durante o processo de redação deste Editorial, fomos surpreendidas com as denúncias de assédio e a exoneração do Ministro Silvio Almeida, que estava à frente do Ministério dos Direitos Humanos e Cidadania do Brasil desde janeiro de 2023 ^11^. O caso remete tanto a questões de direito humano e de saúde coletiva quanto a lutas feministas e antirracistas, podendo ser utilizado para exemplificar o potencial dos estudos do subcampo PPGS, em sua interface com outros âmbitos da ciência, para lidar com problemas complexos.

Nesse cenário, com foco na política e na gestão institucional, pesquisas podem contribuir com análises aprofundadas sobre os contextos relacionados às situações de assédio moral ou sexual vividas por mulheres nas organizações, identificando suas repercussões e interações com a sociedade. Tal compreensão permite desvendar as dinâmicas de poder, os interesses envolvidos e os fatores que facilitam ou dificultam a implementação de políticas efetivas. Estudos científicos podem sugerir recomendações para o aprimoramento das práticas internas de prevenção, detecção e resposta a esses casos, que promovam ambientes de trabalho saudáveis e seguros. Além disso, evidenciar a necessidade de transparência e prestação de contas (*accountability*) nos processos de investigação e decisões institucionais, possibilitando que sejam conduzidos de forma ética e justa. A disseminação de informações científicas que apoiem os direitos das vítimas e reforcem as responsabilidades dos gestores públicos também são aspectos fundamentais. Estudos podem, ainda, indicar estratégias intersetoriais de resposta e prevenção, como programas educacionais e campanhas de conscientização, que não só abordem as situações de assédio, mas contribuam para transformar a cultura institucional, reduzindo a ocorrência de violências vividas por mulheres.

Em síntese, o subcampo PPGS tem um papel fundamental na conformação da Saúde Coletiva. Seus desafios e contribuições são centrais para a construção de políticas e sistemas de saúde mais justos, efetivos e democráticos. Maiores avanços na produção científica desse subcampo dependerão da capacidade de seus pesquisadores e profissionais de enfrentar os desafios contemporâneos e promover um diálogo contínuo entre a pesquisa e a prática de gestão, com o objetivo de melhorar as condições de vida e saúde das populações. 

CSP estará sempre aberto a receber suas contribuições!

# Translation: Política, Planificación y Gestión Sanitaria: reflexiones desde la experiencia de CSP

**DOI:** 10.1590/0102-311XES164524

**Authors:** Luciana Dias de Lima, Mônica Martins

**Affiliations:** 1 Escola Nacional de Saúde Pública Sergio Arouca, Fundação Oswaldo Cruz, Rio de Janeiro, Brasil.

Los estudios que tienen como objetivo comprender y contribuir con las políticas, la organización y la gestión de los sistemas y servicios sanitarios constituyen uno de los ejes estructuradores de la Salud Colectiva en el ámbito internacional y nacional. Estos estudios forman parte de una área con una variedad de temas y aplicaciones, de múltiples niveles y dimensiones ^1^, que va desde un ámbito más general con la formulación e implementación de las políticas públicas y configuración de los sistemas sanitarios hasta el ámbito de las relaciones interpersonales que se establecen en los procesos laborales y de asistencia sanitaria. Además, involucra el desarrollo de estrategias para mejorar la equidad en el acceso, uso y efectividad de los servicios sanitarios, y la producción y evaluación de la incorporación de tecnologías y asignación de recursos financieros.

Este campo comenzó a desarrollarse en Brasil a partir de los años 1970, en el contexto de redemocratización del país y creación del Sistema Único de Salud brasileño (SUS, por sus siglas en portugués), que implicó la articulación entre académicos de diferentes campos del saber, profesionales de la salud y movimientos sociales ^2^. Y poco a poco se consolidó como uno de los tres subcampos que conforman la Salud Colectiva -Política, Planificación y Gestión Sanitaria (PPGS, por sus siglas en portugués)-, junto con la Epidemiología y las Ciencias Sociales y Humanas en Salud ^3^.

*Cadernos de Saúde Pública* (CSP) muestra la dinámica y evolución de este subcampo, el cual estuvo presente en los momentos que marcaron sus cuarenta años de publicaciones científicas ^4^. Una mirada a los títulos de 834 artículos que se publicaron entre 2001 y junio de 2024 y que fueron evaluados por editores asociados vinculados a la PPGS revela temas, enfoques y métodos diversos. Si bien se reconoce la posibilidad de relacionar el campo con otros subcampos de la Salud Colectiva ^5^ ^,^ ^6^, en este período predominaron algunos núcleos temáticos, como de la evaluación, de la atención primaria, de los medicamentos, de las políticas de salud, del acceso a la atención de salud, de la atención especializada e información en salud. Además, la implementación de políticas a nivel nacional, regional o local, o que se enfocan en las necesidades de salud, ya sea conocidas o emergentes, refleja la producción científica en CSP.

Sin embargo, las temáticas y enfoques sufrieron variaciones a lo largo del tiempo. Los cambios son resultado de la diversa representación del subcampo en el Equipo Editorial de la revista, además de sus características distintivas, que están presentes en la elección de objetos, referencias teóricas y diseño de los estudios. Entre las peculiaridades de este subcampo ^7^, se destaca la proximidad entre el entorno académico, las políticas e intervenciones sanitarias, así como la influencia de la agenda gubernamental y los problemas que afectan los sistemas y servicios sanitarios en la definición de temas y preguntas de investigación. Existe una preocupación por las causas de los fenómenos investigados y por las repercusiones de las políticas y modelos de asistencia sanitaria sobre ellos. También hay un cierto eclecticismo en la elección de las referencias teóricas y metodológicas, así como la preferencia por períodos recientes para las investigaciones.

En este subcampo se manifiestan desafíos permanentes como el mantenimiento de la relevancia social y política de los estudios, la garantía de la calidad y el rigor metodológico de la investigación, y la promoción de la difusión y aplicación del conocimiento producido, los cuales van más allá de la agenda de salud colectiva de muchos países. Brasil destaca la importancia en la internacionalización de la producción científica al ampliar el diálogo entre diferentes perspectivas epistemológicas, especialmente entre los países del Sur Global.

Otro desafío corresponde a la necesidad de fortalecer aún más la integración entre la investigación académica y la práctica profesional de la gestión de la salud manteniendo la perspectiva crítica y la calidad de las publicaciones científicas. La expansión de los estudios de maestría y doctorado en Brasil refuerza la relevancia de la PPGS en la formación de posgrado *stricto sensu* en Salud Colectiva, debido a la relación que los programas establecen con el entorno de las políticas y servicios de salud. Esta acción involucra formar a investigadores y gestores capaces de moverse entre estos dos ámbitos, contribuyendo a la construcción de políticas de salud y prácticas de gestión que valoren la ciencia por su capacidad de producir conocimiento, pero también sensibles a la realidad local y a las demandas sociales.

Las necesidades sanitarias que resultan de las transformaciones del mundo contemporáneo desafían, en general, la producción científica en Salud Colectiva ^8^. En este contexto, hay temas emergentes para los autores de la PPGS que aún no fueron suficientemente presentados por CSP. Las relaciones entre el Estado, el mercado y la sociedad en la producción de políticas públicas en el intento de dar una respuesta a estos cambios, tan diversos y que afectan de manera desigual a los países y grupos sociales, ilustran un enfoque que se puede tratar en el subcampo. El modo de interactuación entre diferentes marcadores de desigualdad social (como clase, género, sexualidad, raza, etnia, accesibilidad y vivienda), condicionando las necesidades de salud, de acceso, de uso y de resultados de las políticas y servicios sanitarios, constituye otra temática que merece ser profundizada. También están las cuestiones relacionadas con el cambio climático y las emergencias sanitarias, las cuales requieren resiliencia de los sistemas y servicios de salud, además de nuevas formas de anticipación y preparación en favor de la sostenibilidad y la equidad. Para estos temas, la información integral, ágil y confiable es esencial para permitir la producción de evidencia que contribuya a la toma de decisiones, especialmente en tiempos de grandes avances tecnológicos e informáticos ^9^.

Los esfuerzos de estudios de estos temas emergentes requerirán una mayor articulación entre el conocimiento. La apreciación de un enfoque sistémico e interdisciplinario ^10^, tan citado actualmente, demanda la colaboración entre investigadores con diferentes tradiciones y especializaciones en este subcampo, así como los vinculados a la Epidemiología, las Ciencias Sociales y las Humanidades en Salud, y otras áreas afines.

Durante el proceso de elaboración de este editorial, salieron a la luz denuncias de acoso y el despido del Ministro Silvio Almeida, quien era el jefe del Ministerio de Derechos Humanos y Ciudadanía de Brasil desde enero de 2023 ^11^. El caso expone cuestiones de derechos humanos y salud colectiva, así como de las luchas feminista y antirracista, el cual puede utilizarse como un ejemplo del potencial de los estudios en el subcampo de la PPGS, en su interfaz con otras áreas de la ciencia para abordar problemas complejos.

En este escenario, centrados en la política y la gestión institucional, los estudios pueden contribuir a analizar en profundidad los contextos relacionados con situaciones de acoso moral o sexual contra las mujeres en las organizaciones, identificando sus repercusiones e interacciones con la sociedad. Esta comprensión nos permite revelar la dinámica del poder, los intereses involucrados y los factores que facilitan u obstaculizan la implementación de políticas efectivas. Los estudios científicos pueden aportar con recomendaciones para la mejora de las prácticas internas de prevención, detección y respuesta a estos casos para promover un ambiente de trabajo saludable y seguro. Además, resaltan la necesidad de transparencia y rendición de cuentas (accountability) en los procesos de investigación y las decisiones institucionales, permitiendo su realización de manera ética y justa. También es un aspecto fundamental la difusión de información científica que respalde los derechos de las víctimas y reafirme las responsabilidades de los gestores públicos. Los estudios también pueden indicar estrategias de respuesta y prevención intersectoriales, como programas educativos y campañas de sensibilización, que no solo aborden situaciones de acoso, sino que contribuyan a transformar la cultura institucional al reducir la incidencia de la violencia contra la mujer.

En resumen, el subcampo PPGS desempeña un papel clave en la conformación de la Salud Colectiva. Sus desafíos y contribuciones son fundamentales para construir políticas y sistemas de salud más justos, efectivos y democráticos. Los avances en la producción científica de este subcampo dependerán de la capacidad de sus investigadores y profesionales de enfrentar los desafíos contemporáneos y fomentar un continuo diálogo entre la investigación y la práctica de la gestión, con el fin de mejorar las condiciones de vida y de salud de la población.

¡CSP estará siempre abierto a recibir tus contribuciones!
